# 4-Chloro-1-(4-methyl­phenyl­sulfon­yl)-1*H*-pyrrolo[2,3-*b*]pyridine

**DOI:** 10.1107/S1600536809044559

**Published:** 2009-11-07

**Authors:** Roland Selig, Dieter Schollmeyer, Wolfgang Albrecht, Stefan Laufer

**Affiliations:** a Eberhard-Karls-University Tübingen, Auf der Morgenstelle 8, D-72076 Tübingen, Germany; b University Mainz, Duesbergweg 10-14, D-55099 Mainz, Germany; c c-a-i-r biosciences GmbH, Paul-Ehrlich-Strasse 15, 72076 Tübingen, Germany

## Abstract

The crystal structure of the title compound, C_14_H_11_ClN_2_O_2_S, features a three-dimensional network stabilized by π–π inter­actions between the rings of the 4-methyl­phenyl­sulfonyl protecting group [centroid–centroid distance = 3.623 (1) Å]. The 4-methyl­phenyl­sulfonyl ring makes a dihedral angle of 79.60 (6)° with the 4-chloro-1*H*-pyrrolo[2,3-*b*]pyridine unit.

## Related literature

For the synthesis of the title compound, see: Desarbre *et al.* (1997 ▶).
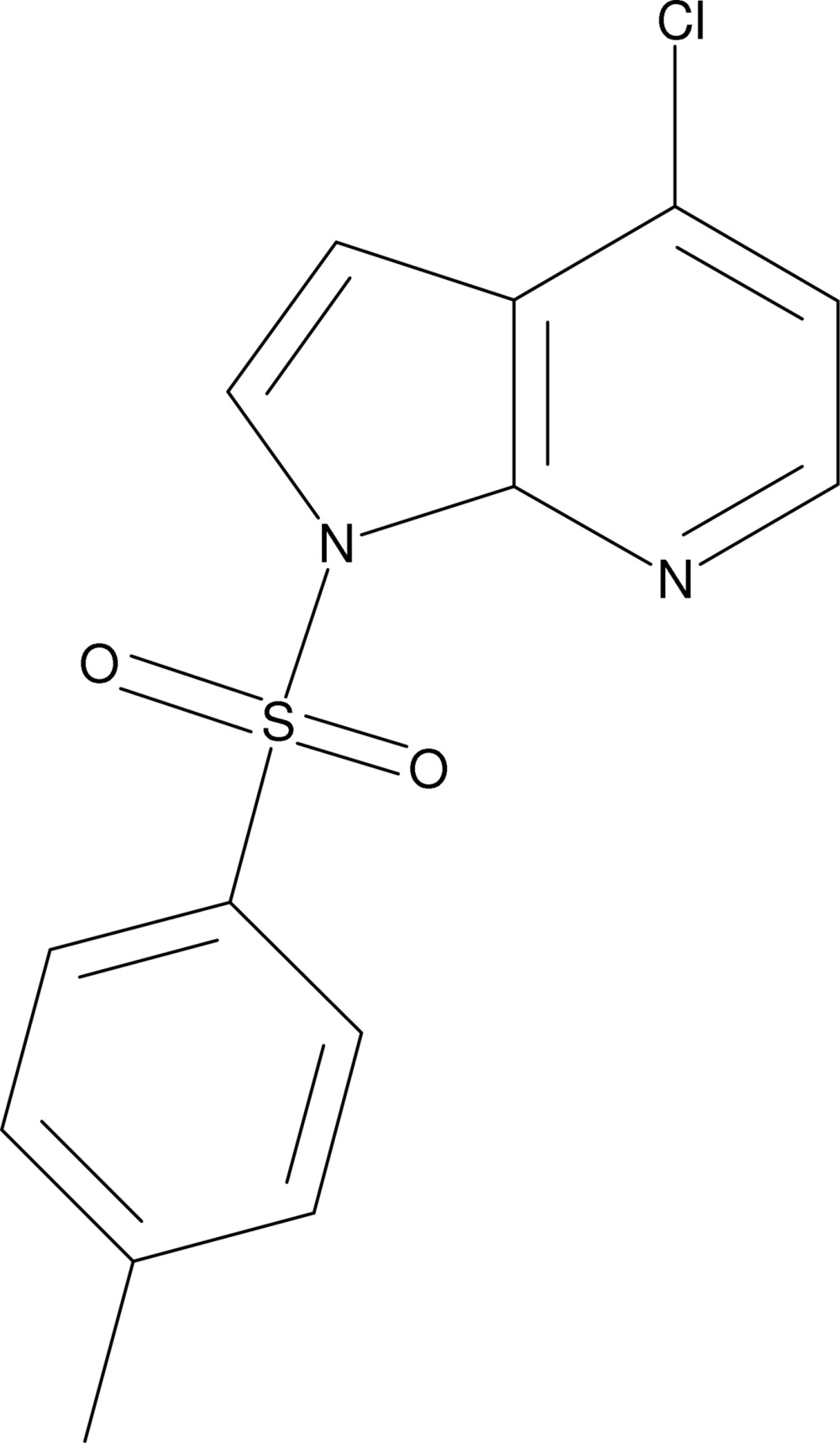


## Experimental

### 

#### Crystal data

C_14_H_11_ClN_2_O_2_S
*M* *_r_* = 306.76
Monoclinic, 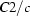

*a* = 21.7342 (12) Å
*b* = 7.6313 (2) Å
*c* = 16.4649 (8) Å
β = 91.531 (2)°
*V* = 2729.9 (2) Å^3^
*Z* = 8
Cu *K*α radiation
μ = 3.94 mm^−1^
*T* = 193 K
0.52 × 0.24 × 0.20 mm

#### Data collection

Enraf–Nonius CAD-4 diffractometer
Absorption correction: numerical (*PLATON*; Spek, 2009 ▶) *T*_min_ = 0.319, *T*_max_ = 0.519
2580 measured reflections
2580 independent reflections
2435 reflections with *I* > 2σ(*I*)
3 standard reflections frequency: 60 min intensity decay: 2%

#### Refinement

*R*[*F* ^2^ > 2σ(*F* ^2^)] = 0.043
*wR*(*F* ^2^) = 0.119
*S* = 1.09
2580 reflections
181 parameters
H-atom parameters constrained
Δρ_max_ = 0.26 e Å^−3^
Δρ_min_ = −0.49 e Å^−3^

### 

Data collection: *CAD-4 Software* (Enraf–Nonius, 1989 ▶); cell refinement: *CAD-4 Software*; data reduction: *CORINC* (Dräger & Gattow, 1971 ▶); program(s) used to solve structure: *SIR97* (Altomare *et al.*, 1999 ▶); program(s) used to refine structure: *SHELXL97* (Sheldrick, 2008 ▶); molecular graphics: *PLATON* (Spek, 2009 ▶); software used to prepare material for publication: *PLATON*.

## Supplementary Material

Crystal structure: contains datablocks I, global. DOI: 10.1107/S1600536809044559/bt5113sup1.cif

Structure factors: contains datablocks I. DOI: 10.1107/S1600536809044559/bt5113Isup2.hkl

Additional supplementary materials:  crystallographic information; 3D view; checkCIF report

## Figures and Tables

**Fig. 1.**
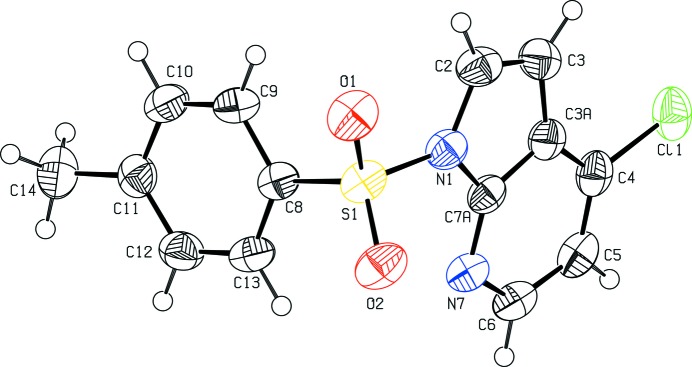
View of compound I. Displacement ellipsoids are drawn at the 50% probability level.

## supplementary crystallographic information

### Comment

In recent years, compounds with the 1*H*-pyrrolo[2,3-*b*]pyridine moiety have been shown to display significant biological activities. The N-protected 4-chloro-1*H*-pyrrolo[2,3-*b*]pyridine is an important precursor for NH sensitive reactions like coupling reactions or metalation experiments. The title compound forms a three dimensional network stabilized by π -π interactions between two phenyl moieties of the 4-methylphenylsulfonyl protecting group (distance between centroids 3.623 (1) Å). The 4-methylphenylsulfonyl ring makes a dihedral angle of 79.60 (6)° to the 4-chloro-1*H*-pyrrolo[2,3-*b*]pyridine.

### Experimental

Finely powdered sodium hydroxide (1.9 g, 34 mmol) was added to a solution of dichloromethane containing benzyltriethylammonium chloride (67 mg, 0.30 mmol) and 4-chlor-1*H*-pyrrolo[2,3-*b*]pyridine (1.5 g, 9.8 mmol). *p*-Toluensulfonylchloride (2.2 g, 12 mmol) was slowly added at 273 K and the resulting suspension was stirred at this temperature for 2 h at room temperature. The suspension was filtered through celite, washed with dichloromethane and the filtrate was evaporated *in vacuo*. The residue was suspendet in methanol and filtered off. The filtrate was dried *in vacuo* to give the pure title compound in a good yield of 78%.

### Refinement

Hydrogen atoms were placed at calculated positions with C_aromatic_—H = 0.95 Å or C_methyl_—H = 0.98Å and they were refined in the riding-model approximation with isotropic displacement parameters (set at 1.2–1.5 times of the *U*_eq_ of the parent atom).

### Crystal data

**Table:**

C_14_H_11_ClN_2_O_2_S	*F*(000) = 1264
*M**_r_* = 306.76	*D*_x_ = 1.493 Mg m^−^^3^
Monoclinic, *C*2/*c*	Cu *K*α radiation, λ = 1.54178 Å
Hall symbol: -C 2yc	Cell parameters from 25 reflections
*a* = 21.7342 (12) Å	θ = 65–70°
*b* = 7.6313 (2) Å	µ = 3.94 mm^−^^1^
*c* = 16.4649 (8) Å	*T* = 193 K
β = 91.531 (2)°	Block, colourless
*V* = 2729.9 (2) Å^3^	0.52 × 0.24 × 0.20 mm
*Z* = 8	

### Data collection

**Table:**

Enraf–Nonius CAD-4 diffractometer	2435 reflections with *I* > 2σ(*I*)
Radiation source: rotating anode	*R*_int_ = 0.0000
graphite	θ_max_ = 69.9°, θ_min_ = 4.1°
ω/2θ scans	*h* = 0→26
Absorption correction: numerical (*PLATON*; Spek, 2009)	*k* = 0→9
*T*_min_ = 0.319, *T*_max_ = 0.519	*l* = −20→20
2580 measured reflections	3 standard reflections every 60 min
2580 independent reflections	intensity decay: 2%

### Refinement

**Table:**

Refinement on *F*^2^	Primary atom site location: structure-invariant direct methods
Least-squares matrix: full	Secondary atom site location: difference Fourier map
*R*[*F*^2^ > 2σ(*F*^2^)] = 0.043	Hydrogen site location: inferred from neighbouring sites
*wR*(*F*^2^) = 0.119	H-atom parameters constrained
*S* = 1.09	*w* = 1/[σ^2^(*F*_o_^2^) + (0.0708*P*)^2^ + 2.2448*P*] where *P* = (*F*_o_^2^ + 2*F*_c_^2^)/3
2580 reflections	(Δ/σ)_max_ < 0.001
181 parameters	Δρ_max_ = 0.26 e Å^−^^3^
0 restraints	Δρ_min_ = −0.48 e Å^−^^3^

### Special details

**Table:**

Geometry. All e.s.d.'s (except the e.s.d. in the dihedral angle between two l.s. planes) are estimated using the full covariance matrix. The cell e.s.d.'s are taken into account individually in the estimation of e.s.d.'s in distances, angles and torsion angles; correlations between e.s.d.'s in cell parameters are only used when they are defined by crystal symmetry. An approximate (isotropic) treatment of cell e.s.d.'s is used for estimating e.s.d.'s involving l.s. planes.
Refinement. Refinement of *F*^2^ against ALL reflections. The weighted *R*-factor *wR* and goodness of fit *S* are based on *F*^2^, conventional *R*-factors *R* are based on *F*, with *F* set to zero for negative *F*^2^. The threshold expression of *F*^2^ > σ(*F*^2^) is used only for calculating *R*-factors(gt) *etc*. and is not relevant to the choice of reflections for refinement. *R*-factors based on *F*^2^ are statistically about twice as large as those based on *F*, and *R*- factors based on ALL data will be even larger.

### Fractional atomic coordinates and isotropic or equivalent isotropic displacement parameters (Å^2^)

**Table:**

	*x*	*y*	*z*	*U*_iso_*/*U*_eq_	
S1	0.37591 (3)	0.21601 (7)	0.24460 (3)	0.04415 (18)	
Cl1	0.17076 (3)	0.02839 (8)	0.52042 (4)	0.0552 (2)	
O1	0.35059 (9)	0.2966 (2)	0.17315 (9)	0.0568 (4)	
O2	0.40887 (9)	0.0548 (2)	0.23960 (10)	0.0574 (4)	
N1	0.31423 (8)	0.1778 (2)	0.30138 (10)	0.0419 (4)	
C2	0.25596 (11)	0.2537 (3)	0.28739 (14)	0.0471 (5)	
H2	0.2448	0.3249	0.2420	0.057*	
C3	0.21846 (10)	0.2106 (3)	0.34773 (14)	0.0452 (5)	
H3	0.1766	0.2440	0.3520	0.054*	
C3A	0.25318 (9)	0.1048 (3)	0.40466 (12)	0.0393 (4)	
C4	0.24293 (10)	0.0231 (3)	0.47865 (13)	0.0428 (5)	
C5	0.29115 (11)	−0.0600 (3)	0.51803 (13)	0.0463 (5)	
H5	0.2853	−0.1165	0.5686	0.056*	
C6	0.34872 (11)	−0.0608 (3)	0.48308 (13)	0.0458 (5)	
H6	0.3814	−0.1178	0.5120	0.055*	
N7	0.36143 (8)	0.0130 (2)	0.41150 (11)	0.0427 (4)	
C7A	0.31332 (9)	0.0897 (2)	0.37609 (12)	0.0374 (4)	
C8	0.41925 (9)	0.3694 (3)	0.30021 (11)	0.0381 (4)	
C9	0.40403 (10)	0.5448 (3)	0.29162 (14)	0.0466 (5)	
H9	0.3696	0.5785	0.2585	0.056*	
C10	0.43905 (10)	0.6698 (3)	0.33118 (14)	0.0478 (5)	
H10	0.4284	0.7899	0.3252	0.057*	
C11	0.48956 (9)	0.6237 (3)	0.37969 (12)	0.0434 (5)	
C12	0.50321 (10)	0.4468 (3)	0.38851 (14)	0.0484 (5)	
H12	0.5373	0.4129	0.4223	0.058*	
C13	0.46853 (10)	0.3189 (3)	0.34933 (13)	0.0451 (5)	
H13	0.4785	0.1985	0.3561	0.054*	
C14	0.52904 (13)	0.7648 (4)	0.41851 (17)	0.0615 (6)	
H14A	0.5628	0.7105	0.4502	0.092*	
H14B	0.5040	0.8362	0.4544	0.092*	
H14C	0.5461	0.8392	0.3761	0.092*	

### Atomic displacement parameters (Å^2^)

**Table:**

	*U*^11^	*U*^22^	*U*^33^	*U*^12^	*U*^13^	*U*^23^
S1	0.0603 (3)	0.0409 (3)	0.0313 (3)	0.0037 (2)	0.0033 (2)	−0.00179 (18)
Cl1	0.0548 (3)	0.0523 (3)	0.0593 (4)	−0.0143 (2)	0.0148 (3)	−0.0159 (2)
O1	0.0779 (11)	0.0603 (11)	0.0319 (7)	−0.0021 (8)	−0.0050 (7)	0.0023 (7)
O2	0.0793 (12)	0.0432 (9)	0.0503 (9)	0.0096 (8)	0.0149 (8)	−0.0083 (7)
N1	0.0500 (10)	0.0398 (9)	0.0359 (8)	0.0004 (7)	−0.0024 (7)	0.0025 (7)
C2	0.0551 (12)	0.0437 (11)	0.0417 (11)	0.0038 (10)	−0.0146 (9)	−0.0013 (9)
C3	0.0437 (11)	0.0434 (12)	0.0479 (11)	−0.0010 (9)	−0.0102 (9)	−0.0115 (9)
C3A	0.0450 (10)	0.0315 (9)	0.0411 (10)	−0.0049 (8)	−0.0031 (8)	−0.0102 (8)
C4	0.0503 (11)	0.0348 (10)	0.0433 (11)	−0.0095 (8)	0.0045 (9)	−0.0113 (8)
C5	0.0645 (13)	0.0360 (10)	0.0383 (10)	−0.0070 (10)	0.0012 (9)	−0.0001 (8)
C6	0.0584 (12)	0.0348 (10)	0.0440 (11)	0.0037 (9)	−0.0036 (9)	0.0026 (9)
N7	0.0509 (10)	0.0355 (9)	0.0416 (9)	0.0038 (7)	0.0007 (7)	0.0006 (7)
C7A	0.0473 (10)	0.0285 (9)	0.0362 (9)	−0.0016 (8)	−0.0013 (8)	−0.0044 (7)
C8	0.0450 (10)	0.0387 (10)	0.0310 (9)	0.0052 (8)	0.0077 (7)	0.0022 (7)
C9	0.0504 (12)	0.0422 (11)	0.0469 (11)	0.0105 (9)	−0.0042 (9)	0.0061 (9)
C10	0.0551 (12)	0.0367 (11)	0.0519 (12)	0.0071 (9)	0.0051 (10)	0.0053 (9)
C11	0.0431 (10)	0.0476 (12)	0.0401 (10)	−0.0026 (9)	0.0107 (8)	0.0034 (9)
C12	0.0431 (11)	0.0534 (13)	0.0483 (12)	0.0063 (10)	−0.0025 (9)	0.0077 (10)
C13	0.0505 (11)	0.0402 (11)	0.0447 (11)	0.0120 (9)	0.0038 (9)	0.0061 (9)
C14	0.0609 (14)	0.0572 (15)	0.0665 (16)	−0.0147 (12)	0.0030 (12)	0.0037 (12)

### Geometric parameters (Å, °)

**Table:**

S1—O1	1.4250 (16)	C6—H6	0.9500
S1—O2	1.4269 (17)	N7—C7A	1.320 (3)
S1—N1	1.6803 (18)	C8—C13	1.380 (3)
S1—C8	1.746 (2)	C8—C9	1.385 (3)
Cl1—C4	1.730 (2)	C9—C10	1.374 (3)
N1—C7A	1.402 (3)	C9—H9	0.9500
N1—C2	1.406 (3)	C10—C11	1.386 (3)
C2—C3	1.343 (3)	C10—H10	0.9500
C2—H2	0.9500	C11—C12	1.389 (3)
C3—C3A	1.436 (3)	C11—C14	1.508 (3)
C3—H3	0.9500	C12—C13	1.382 (3)
C3A—C4	1.392 (3)	C12—H12	0.9500
C3A—C7A	1.406 (3)	C13—H13	0.9500
C4—C5	1.373 (3)	C14—H14A	0.9800
C5—C6	1.391 (3)	C14—H14B	0.9800
C5—H5	0.9500	C14—H14C	0.9800
C6—N7	1.342 (3)		
			
O1—S1—O2	120.52 (10)	N7—C7A—N1	124.77 (19)
O1—S1—N1	103.77 (10)	N7—C7A—C3A	128.40 (19)
O2—S1—N1	106.95 (10)	N1—C7A—C3A	106.81 (17)
O1—S1—C8	109.55 (10)	C13—C8—C9	120.6 (2)
O2—S1—C8	110.09 (10)	C13—C8—S1	121.32 (17)
N1—S1—C8	104.58 (9)	C9—C8—S1	118.05 (16)
C7A—N1—C2	107.91 (18)	C10—C9—C8	119.7 (2)
C7A—N1—S1	126.97 (15)	C10—C9—H9	120.2
C2—N1—S1	124.46 (16)	C8—C9—H9	120.2
C3—C2—N1	109.85 (19)	C9—C10—C11	121.2 (2)
C3—C2—H2	125.1	C9—C10—H10	119.4
N1—C2—H2	125.1	C11—C10—H10	119.4
C2—C3—C3A	107.59 (19)	C10—C11—C12	118.1 (2)
C2—C3—H3	126.2	C10—C11—C14	119.7 (2)
C3A—C3—H3	126.2	C12—C11—C14	122.1 (2)
C4—C3A—C7A	115.35 (19)	C13—C12—C11	121.7 (2)
C4—C3A—C3	136.9 (2)	C13—C12—H12	119.2
C7A—C3A—C3	107.75 (19)	C11—C12—H12	119.2
C5—C4—C3A	118.8 (2)	C8—C13—C12	118.8 (2)
C5—C4—Cl1	120.78 (17)	C8—C13—H13	120.6
C3A—C4—Cl1	120.37 (17)	C12—C13—H13	120.6
C4—C5—C6	119.4 (2)	C11—C14—H14A	109.5
C4—C5—H5	120.3	C11—C14—H14B	109.5
C6—C5—H5	120.3	H14A—C14—H14B	109.5
N7—C6—C5	124.8 (2)	C11—C14—H14C	109.5
N7—C6—H6	117.6	H14A—C14—H14C	109.5
C5—C6—H6	117.6	H14B—C14—H14C	109.5
C7A—N7—C6	113.26 (19)		
			
O1—S1—N1—C7A	175.64 (17)	C2—N1—C7A—C3A	3.3 (2)
O2—S1—N1—C7A	47.22 (19)	S1—N1—C7A—C3A	174.17 (14)
C8—S1—N1—C7A	−69.55 (19)	C4—C3A—C7A—N7	−2.3 (3)
O1—S1—N1—C2	−14.9 (2)	C3—C3A—C7A—N7	175.68 (19)
O2—S1—N1—C2	−143.28 (18)	C4—C3A—C7A—N1	179.38 (16)
C8—S1—N1—C2	99.95 (18)	C3—C3A—C7A—N1	−2.6 (2)
C7A—N1—C2—C3	−2.7 (2)	O1—S1—C8—C13	−151.45 (17)
S1—N1—C2—C3	−173.92 (15)	O2—S1—C8—C13	−16.72 (19)
N1—C2—C3—C3A	1.0 (2)	N1—S1—C8—C13	97.86 (17)
C2—C3—C3A—C4	178.3 (2)	O1—S1—C8—C9	26.4 (2)
C2—C3—C3A—C7A	1.0 (2)	O2—S1—C8—C9	161.17 (17)
C7A—C3A—C4—C5	1.5 (3)	N1—S1—C8—C9	−84.25 (18)
C3—C3A—C4—C5	−175.7 (2)	C13—C8—C9—C10	1.2 (3)
C7A—C3A—C4—Cl1	−179.07 (14)	S1—C8—C9—C10	−176.68 (17)
C3—C3A—C4—Cl1	3.8 (3)	C8—C9—C10—C11	0.0 (3)
C3A—C4—C5—C6	−0.1 (3)	C9—C10—C11—C12	−1.2 (3)
Cl1—C4—C5—C6	−179.55 (16)	C9—C10—C11—C14	176.6 (2)
C4—C5—C6—N7	−0.9 (3)	C10—C11—C12—C13	1.1 (3)
C5—C6—N7—C7A	0.3 (3)	C14—C11—C12—C13	−176.7 (2)
C6—N7—C7A—N1	179.39 (18)	C9—C8—C13—C12	−1.3 (3)
C6—N7—C7A—C3A	1.4 (3)	S1—C8—C13—C12	176.54 (17)
C2—N1—C7A—N7	−175.14 (19)	C11—C12—C13—C8	0.1 (3)
S1—N1—C7A—N7	−4.2 (3)		

## References

[bb1] Altomare, A., Burla, M. C., Camalli, M., Cascarano, G. L., Giacovazzo, C., Guagliardi, A., Moliterni, A. G. G., Polidori, G. & Spagna, R. (1999). *J. Appl. Cryst.* **32**, 115–119.

[bb2] Desarbre, E., Coudret, S., Meheust, C. & Merour, J.-Y. (1997). *Tetrahedron*, **53**, 3637–3648.

[bb3] Dräger, M. & Gattow, G. (1971). *Acta Chem. Scand.* **25**, 761–762.

[bb4] Enraf–Nonius (1989). *CAD-4 Software*. Enraf–Nonius, Delft, The Netherlands.

[bb5] Sheldrick, G. M. (2008). *Acta Cryst.* A**64**, 112–122. 10.1107/S0108767307043930 18156677

[bb6] Spek, A. L. (2009). *Acta Cryst.* D**65**, 148–155. 10.1107/S090744490804362X PMC2631630 19171970

